# Hypertrophic and Keloid Scar Management: Advances in Diagnosis, Perioperative Care, and Anesthetic Modulation

**DOI:** 10.7759/cureus.88810

**Published:** 2025-07-26

**Authors:** Sarah Kazemeini, Ahmed Nadeem-Tariq, Parisa Hajian, Bettina Anil, Jennifer Easterly, Kiratpreet Sraa, Sahara Pokharel, Rebecca Metellus, Monia Kazemeini

**Affiliations:** 1 Medicine, Kirk Kerkorian School of Medicine at the University of Nevada, Las Vegas, Las Vegas, USA; 2 Otolaryngology - Head and Neck Surgery, Kirk Kerkorian School of Medicine at the University of Nevada, Las Vegas, Las Vegas, USA; 3 Dermatology, Arizona College of Osteopathic Medicine, Glendale, USA; 4 Medicine, William Carey University College of Osteopathic Medicine, Hattiesburg, USA; 5 Medicine, Medical College of Georgia at Augusta University, Augusta, USA; 6 Medicine, DeBusk School of Osteopathic Medicine, Knoxville, USA; 7 Medicine, Geisinger Commonwealth School of Medicine, Scranton, USA; 8 Mechanical Engineering, University of Nevada, Las Vegas, Las Vegas, USA

**Keywords:** anesthesia, dermatologic surgery, hypertrophic scars, keloids, perioperative care, scar management, wound healing

## Abstract

Hypertrophic and keloid scars present significant challenges in surgical recovery and perioperative care, especially in patients undergoing reconstructive procedures or burn treatment. These scars can cause pain, pruritus, and psychosocial distress. There is a higher burden in individuals with skin of color, a term commonly used to describe those with Fitzpatrick skin types IV to VI, due to both increased incidence and limitations of early detection. Traditional treatment approaches often overlook the complex nature of wound healing and the influence of perioperative inflammation and pain control. Recent advances, such as high-frequency ultrasound, shear wave elastography, and artificial intelligence tools, have improved the ability to assess scars objectively and detect changes early. Wearable monitoring systems and personalized therapies are also creating new options to guide treatment. Intraoperative and postoperative strategies, including silicone sheeting and corticosteroid injections, are central to improving outcomes. Anesthesiologists contribute by using regional techniques that reduce inflammation and support recovery. This review explores how surgical, anesthetic, and dermatologic strategies together can improve care and reduce disparities in scar management.

## Introduction and background

Abnormal scarring, including hypertrophic scars and keloids, presents a persistent clinical challenge that affects aesthetic outcomes, functional mobility, and quality of life [1-3]. They often result from dysregulated wound healing marked by fibroblast overactivity, excessive collagen deposition, and prolonged inflammation [4]. They can lead to chronic pain, pruritus, and contractures that interfere with daily activity and emotional well-being.

Patients with skin of color face an added burden due to a higher prevalence of pathological scarring and reduced diagnostic sensitivity of early detection tools [5]. Clinical features such as erythema and vascular changes are often underappreciated in darker skin tones, which can delay intervention [6]. Most diagnostic and treatment protocols were not designed with diverse skin types in mind [6]. As a result, these limitations often contribute to poorer outcomes [7].

Recent advances in imaging, molecular profiling, and AI-assisted tools offer the potential for earlier detection, improved risk stratification, and individualized treatment planning. At the same time, perioperative factors such as pain control and inflammation modulation, often managed by anesthesiologists, play a critical role in wound healing and scar outcomes [8]. Technologies such as high-frequency ultrasound, shear wave elastography, and thermal imaging may allow more objective assessment of scar behavior [8,9]. Wearable wound-monitoring systems and bioengineered dermal substitutes are also reshaping prevention and treatment strategies [10]. Despite these promising developments, there remains a critical need for evidence-based approaches to scar assessment and management.

This narrative review synthesizes current strategies in scar mitigation, emerging diagnostic tools, and patient-specific considerations to inform future research and clinical care. A structured literature search was conducted in PubMed, Scopus, and Google Scholar using the following keywords and Medical Subject Headings (MeSH) terms: “hypertrophic scar,” “keloid,” “scar diagnosis,” “skin of color,” “high-frequency ultrasound,” “infrared imaging,” “elastography,” “wound monitoring,” “TGF-β,” and “molecular profiling.” Articles were screened for relevance based on whether they addressed diagnostic challenges, imaging modalities, or molecular profiling in the context of abnormal scar formation. Priority was given to original studies, systematic reviews, and meta-analyses. Animal-only studies and non-English publications were excluded. This review highlights interdisciplinary approaches. including surgical timing, anesthetic modulation, and dermatologic therapies, to provide a more outcome-driven strategy for scar management.

## Review

Pathophysiology of keloid and hypertrophic scar formation

Wound healing is a multi-phase process that includes hemostasis, inflammation, proliferation, and remodeling. Immediately after skin injury, exposed subendothelial structures like collagen and tissue factor activate platelets, which then aggregate and initiate clot formation via the coagulation cascade to minimize blood loss. These platelets release various growth factors and cytokines, which act as chemoattractants for immune cells, such as neutrophils and macrophages, to remove pathogens and cellular debris at the wound site [11]. As healing progresses, growth factors also recruit fibroblasts. Fibroblasts contribute to new tissue formation by producing collagen and extracellular matrix (ECM) and promoting angiogenesis [12]. During the remodeling phase, matrix metalloproteinases (MMPs) help degrade immature ECM and type III collagen [13]. Burn injuries disrupt normal healing by sustaining inflammation and impairing remodeling. The prolonged inflammatory response caused by severe burns often disrupts proper remodeling due to persistent pro-inflammatory cytokine release and extensive tissue destruction [13]. This makes burn patients especially vulnerable to an increased risk of systemic infection and sepsis [14,15].

At the cellular level, pathological scars such as keloids and hypertrophic scars arise from dysregulated fibroblast activity and impaired ECM turnover [16]. In these scars, fibroblasts are overly active and produce excess type I and III collagen, which accumulates in a disorganized manner [16]. This is driven by elevated levels of transforming growth factor-beta (TGF-β) and vascular endothelial growth factor. Increased TGF-β signaling not only promotes collagen synthesis but also suppresses MMP activity, preventing matrix breakdown and promoting scar tissue accumulation [16-20]. Additionally, keloid fibroblasts show resistance to apoptosis, which allows them to survive longer and continue producing ECM components [16].

Clinically, distinguishing between keloids and hypertrophic scars is important but can be challenging. While they both show exaggerated healing responses, they behave differently. Keloids grow beyond the boundaries of the original wound and have a high recurrence rate [17]. In contrast, hypertrophic scars remain confined to the wound margins and often improve over time. Histologically, keloids show dense collagen bundles, while hypertrophic scars feature more organized collagen deposition [21-23]. Recognizing the differences between these scar types is essential for guiding treatment. Keloids often require a combination of therapies, such as surgical excision, corticosteroid injections, and radiation, while hypertrophic scars may respond well to less intensive interventions like silicone sheeting [21]. Early differentiation can be complicated by overlapping features during initial scar maturation [12].

Several factors contribute to the development of pathological scars following burns. Deeper burns cause greater tissue damage and trigger prolonged inflammation, which increases fibroblast activation and collagen deposition. Infections can further exacerbate the condition by elevating inflammatory cytokines [24]. Similarly, delayed wound closure extends the inflammatory phase, providing a sustained stimulus for fibrosis. Genetic predisposition also plays a major role. Individuals of African, Asian, and Hispanic descent are more likely to develop keloids [25]. Additionally, areas of the body exposed to high mechanical tension, such as the chest, shoulders, and upper back, are more susceptible to abnormal scar formation [26-28]. Recognizing these factors allows clinicians to identify high-risk patients and implement preventive measures early in burn care to mitigate the development of pathologic scars.

Diagnostic challenges and emerging tools

Differentiating normal healing from pathological scarring remains a significant clinical challenge. An imbalance in the expression of MMPs has been implicated in various pathological conditions. In abnormal healing, MMP expression levels are often decreased compared to normal cells, disrupting connective tissue remodeling [29]. While superficial epidermal injuries tend to heal efficiently, deeper wounds are more likely to develop into abnormal scars [30]. Despite these insights, predictive tools for identifying patients at risk of keloid formation remain limited. Recent models suggest that factors such as keloid activity assessment scales, blood pressure levels, postoperative complications, and inflammatory cell proportions may help stratify risk [31].

Erythema and early vascular changes, commonly used indicators of inflammation, are often less visible in individuals with darker skin tones [31]. Diagnosing vascular anomalies in these populations is more challenging due to their subtle presentation, though tools like clinical photography and dermoscopy have shown utility in improving detection [16]. These limitations create a diagnostic blind spot that may delay appropriate recognition and management of hypertrophic scars and keloids in patients with skin of color.

Emerging imaging technologies offer new pathways for earlier and more accurate detection of pathological scars. High-frequency ultrasound has been used to assess cutaneous scar thickness, although it remains limited in capturing epidermal and total tissue depth [32]. Shear wave elastography, a modality that quantifies tissue stiffness through shear wave velocity, has shown clinical relevance in identifying active keloid lesions [33]. Additionally, infrared thermal imaging provides an objective measure of inflammation and ongoing scar activity, allowing for noninvasive assessment of tissue under both normal and pathological conditions [34].

To further enhance early scar recognition, molecular profiling strategies are being explored. Transforming growth factor-β (TGF-β) has been identified as a central driver in hypertrophic scar development, with elevated levels observed systemically and at burn sites correlating with scar outcomes [35]. Concurrently, remote and continuous wound monitoring technologies are advancing. Multifunctional wound monitoring devices have shown promise in improving healing outcomes, reducing infection rates, and tracking relevant biomarkers throughout the recovery process [36]. These innovations may ultimately reduce healthcare utilization by decreasing the need for hospitalizations, outpatient visits, and laboratory testing.

Surgical approaches

Once identified, early surgical planning becomes key in reducing scar formation. Early surgical intervention strategies can reduce prolonged inflammation and cytokine upregulation that delay re-epithelialization in deep burn wounds [37]. Though some degree of inflammation is necessary for wound healing, aberrant inflammatory pathways can contribute to hypertrophic scarring. Excision within the first 72 hours after injury reduces the long-term risk for hypertrophic scarring in comparison to excision performed up to two weeks later [38]. Minimizing time to treatment is valuable in achieving the most optimal cosmetic and functional outcomes for patients with burn injuries. 

Following excision, different graft types are used to achieve the optimal recovery and desirable cosmetic outcome. Full-thickness grafts have lower occurrences of secondary contraction, epidermal hyperplasia, and dermal fibrosis than split-thickness grafts [39]. Split-thickness grafts offer improved cosmetic outcomes with reduced erythema and hyperpigmentation, though these complications are not eliminated by either graft choice. 

Due to challenges with the availability of healthy donor skin and contraction development, dermal substitutes such as Integra and Poreskin have also been investigated [40]. Future investigations into the effectiveness of various dermal substitutes as alternatives for grafting are indicated. In more high-risk areas such as the neck, axilla, hand, and knee, where graft contracture is most prominent, special techniques for grafting are indicated to reduce the tension on the skin [39].

Postoperative strategies

The use of adjuvant therapy is recommended to minimize the risk of keloids and hypertrophic scarring. Constant pressure therapy, such as pressure garments, shows regression of hypertrophic scarring by limiting collagen buildup [41]. Topical silicone gel sheeting, a therapy used for over 30 years, is a favorable option for areas with increased motility, such as the neck, axilla, hand, and knee, where pressure garments may not fit properly [42]. Additionally, intralesional steroid injections provide benefits by completely flattening immature raised scars or softening mature scars to some extent [42]. Side effects of dermal atrophy, telangiectasia, and pain at the site of injection have been reported but can be managed by the use of local anesthetic around the wound site [43]. Another option includes onion extract-based gels, which have been shown to reduce scar pigmentation and height in some studies, though results are mixed and benefits are generally modest [44]. The efficacy of these postoperative strategies has been well established, with minor side effects to be noted.

Table 1 summarizes commonly used postoperative therapies for pathological scarring, incorporating findings from randomized trials, expert consensus, and author interpretation.

**Table 1:** Comparison of postoperative therapies for pathological scarring Data adapted from Zhang et al. [41], Atiyeh et al. [42], Manuskiatti et al. [43], and Draelos et al. [44]. Some entries were directly extracted from study findings (e.g., randomized controlled trials or expert consensus), while others were modified based on the author’s interpretation to enhance clinical relevance; no direct reproduction.

Therapy	Mechanism of Action	Ideal Use Cases	Limitations and Considerations
Pressure garments [41]	Mechanical compression reduces collagen deposition	Large surface areas, early intervention	Poor fit on joints, poor compliance
Silicone sheeting [42]	Occlusive barrier modulates hydration and collagen	Mobile areas like the neck, axilla, joints	May peel, difficult in hot climates
Intralesional steroids [43]	Reduces fibroblast activity and inflammation	Raised scars, keloids	Atrophy, telangiectasia, injection pain
Onion extract gel [44]	Inhibits fibroblast proliferation, mild anti-inflammatory	Superficial or early hypertrophic scars	Limited evidence, results variable, over-the-counter

Perioperative and anesthetic considerations

Anesthesiologists play a critical role in the perioperative care of scar-prone patients. Patients who are considered at higher risk for pathological scarring include those with a history of keloids or hypertrophic scars, Fitzpatrick skin types IV to VI, or those undergoing procedures in high-tension areas [6]. Their role begins even before the first incision. For patients with a known history of keloids or hypertrophic scars, early multidisciplinary planning that includes anesthesia can help inform intraoperative decisions. This may include minimizing skin tension and ensuring pain control. Regional anesthesia provides effective pain relief and may reduce the systemic inflammatory response associated with surgery. While not clinically established, some experimental studies have explored the potential anti-fibrotic effects of local anesthetics like lidocaine on dermal fibroblasts in vitro, suggesting possible biologic roles beyond analgesia [45]. This is significant, as persistent inflammation has been linked to increased fibroblast activation and excess collagen deposition [45,46].

Local anesthetics such as lidocaine may also exert anti-fibrotic effects beyond their analgesic function. Experimental studies have shown that lidocaine can inhibit fibroblast migration and downregulate the expression of profibrotic cytokines like TGF-β [46]. In the postoperative setting, well-managed pain can also influence outcomes. Intravenous lidocaine has demonstrated anti-inflammatory properties in some studies, though its relevance to anesthetic technique remains limited [47]. These findings highlight a growing interest in the broader biologic effects of anesthetic agents beyond pain control. Silicone-based dressings and intralesional corticosteroids have been explored as strategies to reduce the likelihood of pathological scar formation in patients with a history of keloids or hypertrophic scarring [48].

Taken together, these perioperative considerations, including preoperative identification of scar-prone patients, use of regional anesthesia to reduce systemic inflammation, and strategies to minimize skin tension, reinforce the value of including anesthesiology in the broader care plan. Through thoughtful anesthetic choices and proactive pain management, clinicians may be able to modulate the biologic processes that lead to disfiguring and symptomatic scars [47,48].

Dermatologic options

Laser therapies have been investigated for their effects on keloid scarring, with the focus centering on the pulsed dye laser (PDL) and CO₂ laser. The PDL laser causes photothermolysis, resulting in hypoxic conditions within the scar [49]. It decreases fibroblast proliferation, allowing for a less dense collagen appearance. Overtreatment can have adverse effects, making two to six treatments ideal for improving scar color, height, and texture [50]. Side effects of erythema and hyperpigmentation remain for this alternative laser therapy. By paralyzing neighboring muscles, it reduces tension and fibroblast activity in the area surrounding a wound [51].

Because tension across a healing wound increases the risk of developing keloid and hypertrophic scars, botulinum toxin could be a beneficial addition to a treatment regimen. Another therapy option is cryotherapy, which shrinks small keloids but results in possible hypopigmentation [52]. 

Surgical, postoperative, and dermatologic therapies can be used alone or in combination to optimize results for patients with hypertrophic scarring or keloid scarring as a sequela of burns. Regardless of the intervention approach, therapy plans should be tailored to the individual based on scar type, location, and skin tone.

Innovations and future directions

Bioengineered skin represents a transformative advancement in burn care by shifting the healing process toward regeneration. Unlike traditional grafts, which often result in collagen deposition and contractures, bioengineered skin grafts are designed to mimic the structure and function of native skin. Incorporating induced pluripotent stem cell-derived keratinocytes and fibroblasts into these bioengineered grafts may further enhance regenerative potential by modulating inflammation and secreting growth factors that suppress profibrotic signals like TGF-β [53]. These grafts can also be enriched with bioactive molecules like vascular endothelial growth factor and hyaluronic acid to accelerate angiogenesis and re-epithelialization while reducing infection risk, an important factor in preventing chronic inflammation [53].

Biomarkers also hold significant potential for predicting pathological scarring. A computational analysis of 120,000 simulated wound-healing scenarios identified specific protein biomarkers, most notably interleukin-10, tissue inhibitor of metalloproteinase-1, and fibronectin, as highly predictive of hypertrophic scar formation. Fibronectin alone achieved a predictive accuracy of 86%, while a combined panel of IL-10, TIMP-1, and fibronectin reached 89% predictive accuracy [54]. These findings highlight the need for time-sensitive markers whose levels change meaningfully during the early phases of inflammation and proliferation. By enabling clinicians to classify patients based on their risk of hypertrophic scarring, biomarker panels could guide the early application of therapies.

3D imaging and AI tools are reshaping the way scars are monitored and are offering quantitative alternatives to traditional visual scales. Conventional methods like the Vancouver Scar Scale rely heavily on subjective evaluation factors that are difficult to assess accurately in pigmented skin tones [5]. Recent advances have introduced the use of smartphone-captured 2D images and color clustering algorithms to segment and track keloid lesions over time [55]. These systems can quantify changes in scar size and pigmentation. AI-powered 3D imaging and histological tools are being used to assess deeper structural features like collagen density and fiber orientation [56]. The integration of these technologies into wearable sensors and mobile apps could significantly enhance scar monitoring, allowing patients to track healing progress and respond to treatment regimens from home. This patient-driven data collection would improve early detection of abnormal scarring and address disparities in access to dermatologic care. By enabling personalized monitoring, AI holds the potential to improve outcomes for diverse patient populations.

Clinical recommendations

The risk associated with the formation of keloids and hypertrophic scars in burn patients is significant and can lead to functional impairments and psychosocial burden, and overall can impact the quality of life for burn survivors. To prevent these complications, early identification and prompt treatment, especially within the first three to six months, are critical for drastic improvement outcomes [57]. There are a variety of factors that contribute to the creation of a treatment plan for the management of scar formation. Key factors include burn severity, location, skin pigment, and genetic risk factors [57].

The process of scar formation begins with surgical wound closure, making closure techniques critical. The method of closure can significantly influence long-term outcomes regarding both cosmetic and functional results. Surgeons should utilize low-tension local flaps as well as subcutaneous suturing techniques that promote naturally raised wound edges that result in minimal cutaneous tension [58]. These efforts minimize the tension on the skin. Additionally, compression therapy can help stabilize the wound and physically inhibit vascular signals for inflammation that can lead to pathologic scar formation [58].

Early interventions are crucial in these therapies and should be prioritized. Delayed intervention can often lead to more unfavorable scarring that may require more aggressive or invasive therapies. Alongside improvement to general scar prevention, it is important to highlight patient populations that may be at increased risk of developing pathological scars. Patients with skin pigmentation at a Fitzpatrick skin type (FST) of IV-VI are at a significantly greater risk for the formation of keloids and hypertrophic scars, yet there is a lack of diversified research regarding these individuals [58]. Thus, the inclusion of diverse populations in clinical research is essential to providing patients with broadly applicable evidence and establishing an equitable standard of care when it comes to scar treatment.

## Conclusions

Hypertrophic and keloid scars represent a persistent clinical challenge in the setting of burn recovery. In these contexts, the risk of exaggerated wound healing is heightened by inflammation, delayed closure, and mechanical tension. These scars can lead to significant pain, restricted range of motion, and lasting psychosocial effects, making an early and accurate diagnosis essential. However, existing diagnostic tools remain limited due to poor sensitivity to early vascular changes and a lack of validated predictive models in diverse populations.

Innovations in scar assessment, including high-frequency ultrasound, shear wave elastography, thermal imaging, and AI-driven 3D modeling, are beginning to bridge this diagnostic gap. Advances in molecular profiling and biomarker discovery also offer the potential to identify at-risk patients before visible scar formation occurs. Novel approaches such as bioengineered skin grafts are redefining the possibilities for both prevention and treatment.

Going forward, a truly effective approach to pathological scarring must prioritize early recognition, integrate personalized risk stratification, and account for the diverse presentations of scar pathology across skin types. Broadening the scope of clinical trials and diagnostic validation to include patients with darker skin tones is critical. With continued innovation and intentional inclusion of diverse skin types in research, the future of scar management can shift from reactive treatment to proactive, patient-specific care.
